# The gap between prevalence of primary dysmenorrhea and available treatment strategies

**DOI:** 10.1530/RAF-25-0103

**Published:** 2026-03-18

**Authors:** Laasya Koduri, Shivani Anandhasenthil, Lisa Zou, Annmarie Vilkins, Ripla Arora

**Affiliations:** ^1^ Department of Obstetrics, Gynecology and Reproductive Biology, Michigan State University, East Lansing, Michigan, USA; ^2^ Institute for Quantitative Health Science and Engineering, Michigan State University, East Lansing, Michigan, USA; ^3^ Department of Biomedical Engineering, Michigan State University, East Lansing, Michigan, USA; ^4^ Henry Ford Health, Department of Women’s Health Services, Detroit, Michigan, USA

**Keywords:** dysmenorrhea, prostaglandin F2-alpha, non-steroidal anti-inflammatory drugs, oral contraceptive pills, oxytocin

## Abstract

**Abstract:**

Primary dysmenorrhea is the cramping pain in the lower abdomen usually before or during menstruation. Although dysmenorrhea affects young girls and women of all ages, the etiology and treatment options are sparse and have limitations. Through a review of the existing literature, we document the history and elaborate on the mechanisms, diagnosis, and treatments related to dysmenorrhea. Based on cross-sectional studies, primary dysmenorrhea is a common issue affecting many women of reproductive age, often leading to absenteeism, and interfering with daily activities. Treatments such as non-steroidal anti-inflammatory drugs and oral contraceptives have been developed; however, they are not always effective, highlighting a gap between societal needs and available treatment options. To uncover the mechanisms underlying painful contractions, *in vitro* systems and animal models have been used, but they have limitations and do not fully replicate the condition, highlighting the gap between the prevalence of dysmenorrhea and our understanding of the etiology of dysmenorrhea and treatment options. A better understanding of the mechanisms that regulate uterine contractions through development of new model systems is needed to identify novel treatment approaches for dysmenorrhea.

**Lay summary:**

Primary dysmenorrhea or menstrual cramps are a common issue affecting many women of reproductive age, often leading to absenteeism, and interfering with daily activities. In our review, we highlight that treatments such as non-steroidal anti-inflammatory drugs and oral contraceptives have been developed; however, they are not always effective, highlighting a gap between societal needs and available treatment options. We also note that to uncover the mechanisms underlying painful contractions, *in vitro* systems and animal models have been used, but they have limitations and do not fully replicate the condition. Finally, we suggest that a better understanding of the mechanisms that regulate uterine contractions is needed to identify novel treatment approaches for dysmenorrhea.

## Introduction and history of dysmenorrhea

Primary dysmenorrhea is a leading problem that affects adolescent girls and menstruating women (Hailemeskel *et al.* 2016). The prevalence of primary dysmenorrhea varies depending on the region, ranging from 60% (Canadian study) to 85.4% (Ethiopian study) (Burnett *et al.* 2005, Polat *et al.* 2009, Ortiz 2010, Hailemeskel *et al.* 2016). The American College of Obstetricians and Gynecologists define primary dysmenorrhea as cramping pain in the lower abdomen before or during the menstrual period (ACOG committee opinion no. 760: dysmenorrhea and endometriosis in the adolescent 2018). Although menstrual pain is common, it can be distressing and may interfere with well-being. Studies have shown that many women feel that pain related to dysmenorrhea is not validated, and many healthcare providers show indifference toward dysmenorrhea (Wiggleton-Little 2024). Many women experience frustration, delayed diagnosis, and inadequate or ineffective treatment when trying to receive medical help for dysmenorrhea (Chen *et al.* 2018*a*).

**Table 1:** Effectiveness of clinical treatment options available for primary dysmenorrhea.

Treatment type	Examples	Evidence for effectiveness	Mechanism	Remarks
Non-selective NSAIDs (targeting both COX-1 and COX-2)	Ibuprofen, naproxen, mefenamic acid	Evidence that they reduce menstrual pain by lowering prostaglandin levels. No one drug has been shown to work significantly better than another	Block COX enzymes → reduce prostaglandin production → less uterine contraction and pain	First-choice treatment; inexpensive and effective. Main side effects are stomach irritation and GI upset
Selective COX-2 inhibitors	Celecoxib, valdecoxib, rofecoxib	Studies show pain relief similar to traditional NSAIDs	Specifically block COX-2 → reduce prostaglandins → less uterine contraction and pain	May cause fewer stomach issues but are more expensive and not always approved specifically for period pain. Some have safety concerns
Combined estrogen + progesterone OCPs	Ethinylestradiol + progestin component:	Research shows that OCPs decrease menstrual bleeding and lower dysmenorrhea rates. Different generations of OCPs are comparably effective for dysmenorrhea. Limited evidence for effectiveness specifically in primary dysmenorrhea	Prevents ovulation and thins uterus lining → less endometrial shedding → less prostaglandin release → less uterine contractions → reduced pain	Must be taken daily for effectiveness. Contraindicated in patients trying to get pregnant or who have high-risk medical conditions. Newer-generation OCPs have fewer androgenic effects
	Examples of progestins: Norethindrone acetate (1st gen), Levonorgestrel (2nd gen), Norgestimate (3rd gen), Drospirenone (4th gen)			
Progestin-only options/LNG-IUDs	Levonorgestrel IUD (Mirena®), oral progestins	Research shows that the LNG-IUD reduces menstrual pain and bleeding and may work better than oral contraceptive pills in some cases	Thin the uterine lining → less prostaglandin release → less uterine contraction → reduced pain	Long-term option; especially useful for women who cannot take estrogen. Still underused for primary dysmenorrhea, and more direct comparisons are needed

In ancient times, menstruation was often seen as a magical or supernatural phenomenon, imbuing people of their period with special powers. However, during the medieval period, attitudes toward menstruation shifted, and shame and stigma were imposed on those experiencing it (Grandi *et al.* 2012). Even when individuals experienced pain, they were often denied medication. Unfortunately, in many places today, menstruation is still considered a sign of impurity and uncleanliness, further perpetuating the stigma around dysmenorrhea (Jaafar *et al.* 2023). This stigma has hindered the research and development of effective treatments for menstrual pain.

**Figure 1:**
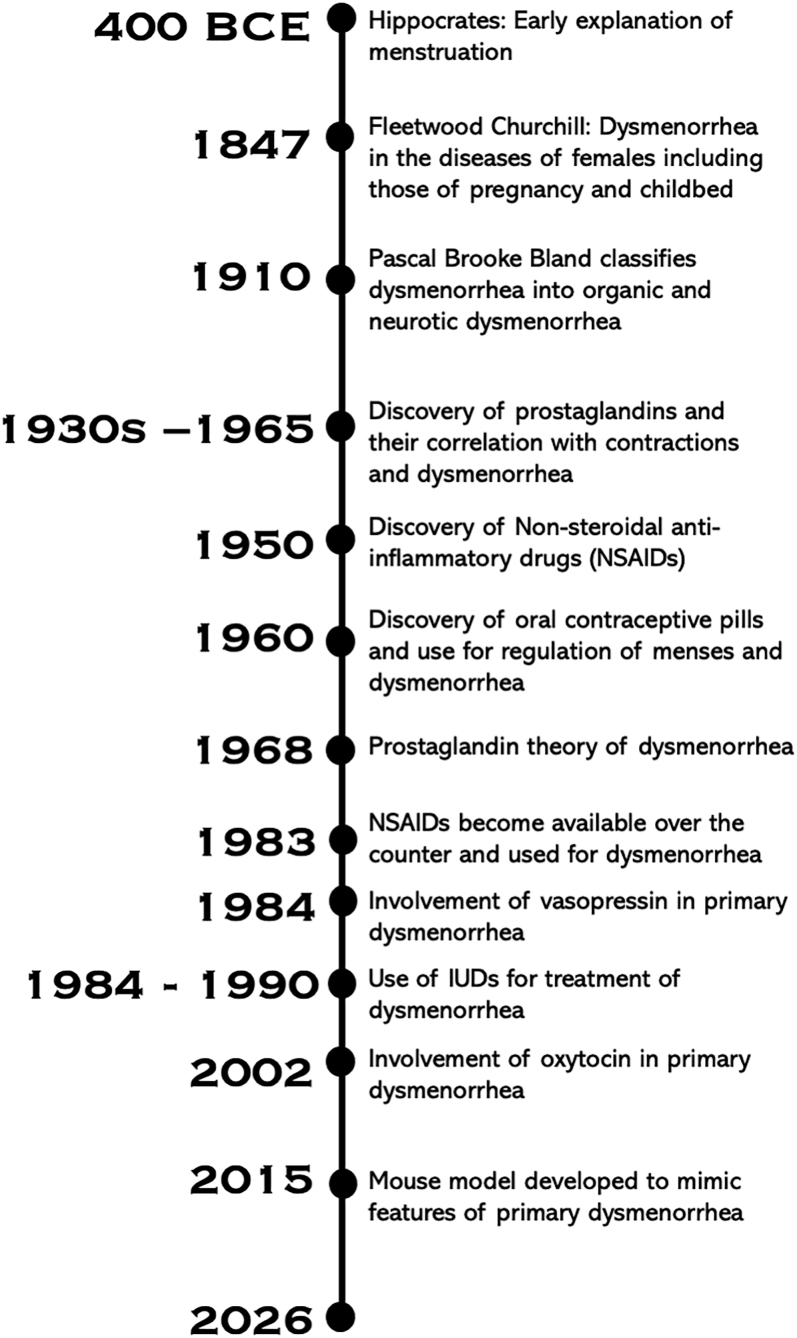
Dysmenorrhea at a glance. A timeline of events related to the etiology, diagnosis, and treatment for dysmenorrhea.

Abnormal menstrual bleeding, including painful menstruation, has been a topic of discussion for thousands of years. One of the earliest recorded explanations for painful menstruation dates back to around 400 BCE (Fig. 1), when the Greek physician Hippocrates theorized that when menstrual blood is not properly discharged, it accumulates, causing discomfort or pain (Santora 2021, Sima *et al.* 2022). This theory emphasized the importance of maintaining balance and proper flow within the body’s humoral system, a fundamental concept in ancient Greek medicine.

During the 19th century, physicians started categorizing painful menstruation as dysmenorrhea. The term ‘dysmenorrhea’ comes from Greek roots – *dys* (painful, difficult), *men* (month), and *rhoia* (flow). Dr William Campbell was among the first physicians to describe menstrual pain clinically (Campbell 1827). In 1847, the English physician Fleetwood Churchill wrote about dysmenorrhea in his book ‘The Diseases of Females: Including Those of Pregnancy and Childbed’ (Fig. 1). He noted that those who suffer from dysmenorrhea often experience persistent and severe pain (Grandi *et al.* 2012). Churchill also described different types of dysmenorrhea, including neuralgic, inflammatory, and mechanical dysmenorrhea (Churchill 1836). Among the treatments he suggested was the insertion of a catheter-like device through the cervix, which would allow for the injection of warm water into the uterus as a means of relieving pain (Churchill & Condie 1857). Churchill’s work represents an early effort to classify and understand dysmenorrhea and to develop treatments to alleviate the suffering of those who experience it. Dr Mary Putnam Jacobi was a pioneering physician and researcher who debunked 19th-century myths that menstruation weakened women. In her 1876 book, ‘The Question of Rest for Women during Menstruation’, she ascribes dysmenorrhea to abnormal activation of nerves, increasing the pressure of the uterine vessels, which eventually results in abnormal uterine contractility (Jacobi 1876).

In 1910, physician Pascal Brooke Bland published an article titled ‘Dysmenorrhea: Its Significance and Treatment’, where he introduced new classifications of dysmenorrhea. Bland described only two types of dysmenorrhea, which he named neurotic dysmenorrhea and organic dysmenorrhea (Santora 2021). During the 1930s, the Swedish researcher Ulf von Euler identified that prostaglandins are hormone-like substances that are produced by the uterus and play a role in triggering contractions of the uterus during menstruation. In 1965, researcher V.R. Pickles affirmed that women who had a higher level of prostaglandins experienced more primary dysmenorrhea (Santora 2021).

As of 2021, physicians continue to recognize the types of dysmenorrhea that Bland introduced. However, the two types now are primary (formerly neurotic) and secondary (formerly organic) dysmenorrhea. Primary dysmenorrhea is defined as an onset closer to pubertal age. Secondary dysmenorrhea is secondary to other conditions, such as endometriosis, adenomyosis, and fibroids, or due to congenital conditions, such as obstructive Müllerian anomalies (2018*a*). These classifications have remained mostly unchanged over the years. This review focuses on primary dysmenorrhea, and a discussion on secondary dysmenorrhea is out of the scope (for information on secondary dysmenorrhea, see Bernardi *et al.* (2017) and Martire *et al.* (2023)).

## Mechanisms underlying dysmenorrhea

### Prostaglandins

Prostaglandins are chemicals that cause the muscles of the uterus to contract, or tighten, during menstruation, causing the endometrium to shed through the vagina and often causing cramps (Rosenwaks & Seegar-Jones 1980). The effectiveness of prostaglandin synthesis inhibitors in the treatment of dysmenorrhea provides strong evidence supporting the ‘prostaglandin theory’, which suggests that the synthesis of prostaglandins leads to uterine contractions (Fig. 1). Prostaglandin F2-alpha (PGF2α) is the specific stable prostaglandin that stimulates the contraction of uterine smooth muscle and produces vasoconstriction within some blood vessels (Powell *et al.* 1985, Nagy *et al.* 2023). On the first day of a period, the levels of prostaglandins are high. In cases of dysmenorrhea, the body may produce more prostaglandins than normal, which causes more contractions and cramps because of the constriction of the blood vessels around the uterus. As bleeding continues and the lining of the uterus is shed, the levels of prostaglandins go down (Schramm *et al.* 1984). This is why pain tends to lessen after the first few days of a period.

### Ischemia

Dysmenorrhea is also thought to be due to blood flow restriction, resulting from increased intrauterine pressure, vessel constriction, and decreased uterine blood flow (Rosenwaks & Seegar-Jones 1980). During menstruation, the uterus contracts to shed the endometrial lining, and these contractions can lead to increased intrauterine pressure. In some women, these contractions may be more intense or prolonged, leading to reduced blood flow to the uterus resulting in tissue ischemia (Maybin & Critchley 2015), and a lack of sufficient oxygen to muscles leads to pain development.

### Oxytocin

Primary dysmenorrhea can be associated with oxytocin. Oxytocin increases the sodium permeability of uterine myofibrils, which therefore indirectly stimulates the contraction of uterine smooth muscle. The contraction results in a reduction in blood flow and is linked to increased pain (Maybin & Critchley 2015).

### Vasopressin

There is conflicting literature on a possible role for vasopressin in primary dysmenorrhea. In one study, women with premenstrual pain had significantly higher levels of vasopressin compared to a control group. Furthermore, in the group with dysmenorrhea, there was a notable increase in the plasma concentration of prostaglandin metabolites (Akerlund 2002). The most intense pain is caused due to the combined effects of vasopressin and PGF2α, causing both increased contractile activity and reduced uterine blood flow (Akerlund 2002). However, another study reports no difference in vasopressin levels in women with and without dysmenorrhea and reports that using an antagonist for vasopressin did not relieve pain (Valentin *et al.* 2000).

Understanding the role of prostaglandins, oxytocin, and vasopressin in dysmenorrhea sheds light on the mechanisms behind menstrual pain. Prostaglandins, particularly PGF2α, contribute to uterine contractions and cramps, while vasopressin and oxytocin further exacerbate discomfort by affecting uterine contractility and blood flow. However, despite this progress, there are still limitations in our understanding of the precise mechanisms controlling uterine contractions. This lack of comprehensive understanding means that while we recognize the roles of these biochemical factors, we have yet to fully grasp their interactions with other potential contributors to menstrual pain.

Primary dysmenorrhea often begins within the first year after menarche, when ovulatory cycles start becoming regular (ACOG committee opinion no. 760: dysmenorrhea and endometriosis in the adolescent 2018). The individual variability in menstrual pain suggests that there may be novel and unknown factors at play. Adolescence is a time of major hormonal change because the hypothalamic–pituitary–ovarian (HPO) axis is still maturing, and early menstrual cycles often have irregular patterns of estrogen and progesterone (Apter 1997, Gordon *et al.* 2008). The nervous system that processes menstrual pain is also still developing; studies in adolescent neurodevelopment show that pain-processing pathways, including cortical and subcortical regions involved in sensory and emotional responses to pain, continue maturing through the teenage years (Fitzgerald 2005). Most of what we know about dysmenorrhea comes from studies in adult women, so there is a clear gap in knowledge of why primary dysmenorrhea pain begins during adolescence. Some studies have followed up girls from pre-menarche to early post-menarche and measured hormones, menstrual pain, and inflammatory markers, showing that pain patterns can change rapidly in the first years after menarche. Because research specifically examining the HPO axis maturation and pain mechanisms during adolescence is limited, more adolescent-centered studies are needed to understand the early onset of dysmenorrhea and to guide targeted prevention and treatment strategies.

## Treatment strategies

Dysmenorrhea has been treated using medications (Table 1), natural treatments, and physical treatments as described below.

### Medications

#### NSAIDs

Non-steroidal anti-inflammatory drugs (NSAIDs), which were discovered around the 1950s (Fig. 1), are the best established initial therapy for dysmenorrhea. NSAIDs have a direct effect of blocking prostaglandin production by inhibiting the action of prostaglandin synthases (PTGS) also known as cyclooxygenase (COX) enzymes responsible for the formation of prostaglandins. The COX enzyme exists in two forms: COX-1 (PTGS1) and COX-2 (PTGS2). COX-1 is constitutively expressed, while COX-2 is high during early days of pregnancy (St-Louis *et al.* 2010). During menstruation, the levels of cyclooxygenase-2 (COX-2) rise and lead to an increase in prostaglandin production, which is essential for uterine contractions and the shedding of the endometrial lining. As the body synthesizes prostaglandins, COX-2 becomes more active to assist in this process. This increased COX-2 activity contributes to the inflammation and pain experienced during menstruation, especially for those with dysmenorrhea (Lai *et al.* 2019).

Traditional NSAIDs, such as naproxen and ibuprofen, inhibit both COX-1 and COX-2 enzymes, making them non-selective. The lack of prostaglandin leads to reduced uterine contractions and reduced menstrual blood loss. A systematic review concluded that NSAIDs did fulfill their purpose. Drugs such as naproxen and ibuprofen are effective in reducing menstrual blood loss (Grimes *et al.* 2006). However, ibuprofen (IBU) results in first-pass effects and gastrointestinal irritation. To bypass these side effects, an IBU-loaded hexagonal liquid crystal gel (IBU HLC) was developed for transdermal administration, which showed that IBU entered blood circulation and then the uterus, and had a certain targeting ability. It was concluded that IBU HLC gel would be a promising sustained-release preparation for transdermal administration to relieve dysmenorrhea with a significant concentration in the uterus (Xia *et al.* 2023).

COX-2-selective inhibitors, such as celecoxib and valdecoxib, offer another option for managing dysmenorrhea. Unlike traditional NSAIDs that block both COX-1 and COX-2, these drugs primarily inhibit COX-2, which helps reduce prostaglandin production with fewer side effects, including gastrointestinal side effects, headaches, and drowsiness (Marjoribanks *et al.* 2015). Clinical studies have shown that celecoxib provides pain relief comparable to ibuprofen and naproxen for primary dysmenorrhea while causing less stomach irritation. However, COX-2 inhibitors still have important limitations (Daniels *et al.* 2005, 2009, Chou *et al.* 2006). Their long-term use has been associated with an increased cardiovascular risk, and they are generally not recommended as first-line therapy. In addition, evidence for COX-2 inhibitors specifically in primary dysmenorrhea is more limited compared to traditional NSAIDs, and more studies are needed to understand which patients benefit most.

Although NSAIDs block prostaglandin production, they do not clear out previously synthesized prostaglandins. Therefore, pain is still experienced if NSAIDs are not taken preemptively. NSAIDs can also harm the liver if taken in large doses since the liver is responsible for the metabolism of drugs in the body. In addition, 18% of patients with dysmenorrhea do not respond to NSAIDs, illustrating the need for more targeted research regarding the impact of individual patient factors on treatment options (Oladosu *et al.* 2018).

#### OCPs

The second choice of treatment is oral contraceptive pills (OCPs), commonly known as birth control, first developed in the 1960s (Proctor *et al.* 2001) (Fig. 1). These pills control menstrual bleeding by preventing ovulation and thinning the lining of the uterus. Prostaglandins are generated when the uterine lining sheds, so when a uterus does not have much tissue to break down, it results in decreased prostaglandin production, which reduces pain. OCPs consist of only progesterone or a combination of both estrogen and progesterone. The combined OCPs (COCPs) are grouped into first, second, third, and fourth generations, which each vary in the type of progesterone (Cooper & Patel 2024). The estrogen component is one of estradiol, ethinylestradiol, estetrol, or mestranol. First-generation COCPs contain norethindrone acetate, ethynodiol diacetate, lynestrenol, or norethynodrel as their progestin component, whereas second-generation COCPs contain levonorgestrel or dl-norgestrel. Both first- and second-generation COCPs lead to more androgenic side effects. Third-generation COCPs (containing norgestimate, gestodene, or desogestrel) and fourth-generation COCPs (containing drospirenone or cyproterone acetate) are newer and have fewer androgenic side effects. Studies have shown no difference between different COCPs when treating dysmenorrhea (Milsom *et al.* 1990). Although COCPs are helpful in treating secondary dysmenorrhea, there is limited information on the efficacy of COCPs in treating primary dysmenorrhea (Casper 2017).

Although OCPs lower the rate of dysmenorrhea in women, they can also lead to side effects. While less common with modern birth control dosing, COCPs contain high levels of estrogen, which can negatively impact the liver (LiverTox 2012). Estrogen-containing OCPs also increase the risk of other significant adverse effects, including venous thromboembolism and ischemic stroke, making them contraindicated for use by patients with high-risk but common medical conditions (Nguyen *et al.* 2024). Progesterone-only pills can be used as a COCP replacement in women who cannot take estrogen. However, OCPs are only effective when taken daily and cannot be taken when the person is trying to get pregnant.

#### LNG-IUDs

Another conventional treatment method that continues to grow is levonorgestrel-releasing intrauterine devices (LNG-IUDs) (Fig. 1). These IUDs are placed in the uterus and release levonorgestrel to prevent pregnancy. Levonorgestrel works like the progesterone component of OCPs to suppress both endometrial growth and prostaglandin production, leading to lighter periods or oftentimes amenorrhea. LNG-IUDs also inconsistently prevent ovulation to decrease bleeding and pain (Dhamangaonkar *et al.* 2015). Randomized controlled trials and meta-analyses show that the LNG-IUD significantly reduces dysmenorrhea severity and improves long-term quality of life (Gemzell-Danielsson *et al.* 2012, Wang *et al.* 2022). A 2024 study found that women using the LNG-IUD had a faster and greater improvement in menstrual pain compared with women using the copper IUD (Ramazanzadeh *et al.* 2012). However, there was no difference in satisfaction or quality of life between these IUDs (Ramazanzadeh *et al.* 2012, Liu *et al.* 2024).

When comparing treatments for primary dysmenorrhea, non-selective NSAIDs and selective COX-2 inhibitors both reduce prostaglandin production, but as discussed earlier, they differ in side effect profiles and accessibility. COCPs reduce menstrual pain by preventing ovulation and thinning the uterine lining, which decreases prostaglandin release, although head-to-head trials of different formulations for pain relief remain limited (Dawood & Khan-Dawood 2007). The LNG-IUD seems to work as well as, and in some studies even better than, other hormonal methods for relieving dysmenorrhea. Compared to COCPs and other progestin-only pills, the LNG-IUD may offer stronger and longer-lasting relief because it provides a steady release of hormone directly in the uterus. Because of its long-lasting effect and low maintenance, the LNG-IUD may be particularly useful for those who cannot tolerate systemic medication. Still, there are not many direct comparisons between the LNG-IUD and other hormonal options in people with only primary dysmenorrhea. Thus, while NSAIDs provide fast, on-demand pain relief, hormonal treatments, especially the LNG-IUD, give long-term control. Yet, not every patient can use or benefit from each option due to side effects, contraindications, or access barriers, highlighting a clear treatment gap and the need for more tailored solutions.

### Natural treatments

Various natural therapeutic modalities have been utilized for the management of dysmenorrhea. There has been a focus on the efficacy of zinc sulfate as a potential intervention. Recent scholarly investigations have elucidated its effectiveness in ameliorating menstrual discomfort, particularly when administered across multiple menstrual cycles (Obiagwu *et al.* 2023).

Vitamin supplementation has also been investigated, specifically with regard to the influence on pain perception and menstrual blood loss. The existing literature attests to the capacity of vitamin D supplementation to effectively alleviate pain associated with dysmenorrhea. Notably, however, the observed effects do not extend to the modulation of menstrual blood loss, indicating that vitamin D primarily addresses the pain dimension of dysmenorrhea (Amzajerdi *et al.* 2023). Emerging evidence has also shown that vitamin E exhibits promise in alleviating symptoms, attributed to its perceived reduction in pain severity and overall enhancement of menstrual well-being (Alikamali *et al.* 2022).

Another research review explored the use of Ge Gen Decoction (GGD), a traditional Chinese herbal remedy, for treating dysmenorrhea. The study used an animal model with hormonally induced dysmenorrhea to see how GGD affected pain and inflammation. The results were positive, showing that GGD reduced pain and had an impact on inflammation in the uterus. Interestingly, when terazosin, a commonly used alpha-blocker, was given alongside GGD, its effects were weakened, highlighting the importance of understanding how GGD’s anti-inflammatory actions work and interact with other substances (Xie & Qian 2023). Tabebuia avellanedae, a plant, could also help with dysmenorrhea. It was demonstrated that an extract from this plant reduced pain significantly and had anti-inflammatory effects in the uterus (McClure *et al.* 2022).

Turmeric roots, known as Curcuma longa, are also a potential treatment for painful periods. Both regular turmeric roots and the ones processed with vinegar have been evaluated. It was observed that while both preparations helped reduce dysmenorrhea symptoms, vinegar processing improved the ability of turmeric to reduce menstrual pain (Peng *et al.* 2023).

There has been limited research to explore the compounds in the natural treatments that lead to pain relief, and thus, the mechanisms underlying natural treatments remain understudied and less understood.

### Physical treatments

Different research studies have examined various physical treatments to alleviate dysmenorrhea, highlighting distinct approaches to addressing this condition. One study focused on the role of myofascial trigger points in abdominal muscles and their potential contribution to abdominal pain in primary dysmenorrhea (Temel & Bağcıer 2023). Furthermore, another research endeavor highlighted the prevalence of myofascial pain syndrome among women with primary dysmenorrhea, particularly in the abdominal and pelvic regions. This underscores the significance of considering muscle pain as a factor in understanding and managing painful periods (Serrano-Imedio *et al.* 2022). Limited investigational studies suggest an improvement in dysmenorrhea with osteopathic manipulative treatment (Matsushita *et al.* 2020, Serrano-Imedio *et al.* 2022). In a separate study, the alignment of the spine and pelvis was investigated in relation to primary dysmenorrhea. The findings suggested that specific angles of spinal and pelvic alignment could influence the severity of pain experienced during menstruation (Wang *et al.* 2023*b*).

Articular acupoint therapy has been shown to be more effective in reducing menstrual pain compared to NSAIDs. This therapy includes acupuncture, electroacupuncture, and acupressure. A review of 11 randomized control trials concluded that articular acupoint therapy can help with primary dysmenorrhea symptoms, regardless of body part or experiment duration, especially in women refraining from taking medications (Cao *et al.* 2023).

Transcutaneous auricular vagus nerve stimulation is a non-invasive technique that electrically stimulates the auricular branch of the vagus nerve in the ear. This stimulus creates a parasympathetic nervous system response, leading to vasodilation in the pelvic organs and reducing ischemia pain. After 10 days of 1 Hz transcutaneous auricular vagus nerve stimulation performed 1–2 days after menstruation, it was concluded that dysmenorrhea symptoms were improved and effects lasted for one menstrual period after stimulation (Wang *et al.* 2023*a*). Transcutaneous electrical neurostimulation (TENS) has also been shown to relieve primary dysmenorrhea symptoms. In a trial with a control and experiment group, after the first two applications of TENS, there was a decrease in pain that sometimes lasted for more than 7 h (Guy *et al.* 2022).

Regardless of medications, physical exercise can efficiently target dysmenorrhea symptoms. A recent study conducted with university students showed that performing high-intensity exercises on a spinning bike downregulated high-sensitivity C-reactive protein, PGE2, and PGF2α, which decreased pain severity and reduced uterine contraction and inflammation (Huang *et al.* 2022). When considering low-intensity workouts, yoga is a good alternative as it can reduce pain intensity and duration for dysmenorrhea. Yoga has been shown to decrease homocysteine levels, an oxidative stress marker induced during dysmenorrhea (Kanchibhotla *et al.* 2023). In a randomized trial, three different groups were assigned a squat variation to perform along with yoga, with a controlled group only practicing yoga. It was concluded that subjects reported less pain when performing squats compared to subjects that did not perform squats. Yoga regulates the stress pathways, which is key in regulating hormonal balance and reducing dysmenorrhea pain (Yosri *et al.* 2022).

Collectively, these diverse studies underscore the multifactorial nature of dysmenorrhea and the subsequent multifaceted nature of physical treatments for it. These investigations contribute to a comprehensive understanding of the varied strategies that can potentially provide relief for individuals suffering from dysmenorrhea. However, similar to natural treatments, the mechanisms by which these treatments reduce symptoms of dysmenorrhea are not well understood. This further highlights how little we know about the mechanisms that cause dysmenorrhea in the first place and that there is an urgent need for extensive research into the etiology of dysmenorrhea.

## Model systems to study dysmenorrhea and uterine contractions

Selecting a model species to study a condition must be done carefully. For studying dysmenorrhea, among many variables, uterus size, number of follicles released per uterine cycle, and hormone control might differ between species (Liechty *et al.* 2015). Non-human primate species, such as the rhesus macaques, present a good model system due to similar reproductive anatomy and physiology of the uterus. However, due to ethical guidelines for macaques, cost, and limited access to macaques, rodents such as mice and rats are frequently chosen to study mechanisms underlying dysmenorrhea.

Rodents provide a good model system because of similarities in the mesodermal origin of the rodent and human uterine muscle and the advanced genetic engineering technologies available to discover targets unique to the reproductive tract (Tuo *et al.* 2023). Because of their similarities to humans regarding vaginal bleeding, spiny mice have been used multiple times as a model system to study menstruation and dysmenorrhea (Liu *et al.* 2020). The spiny mouse undergoes spontaneous decidualization, which allows for the study of mechanisms of menstruation. However, a dysmenorrhea model of spiny mouse has not been generated yet.

### Mouse model of dysmenorrhea

Researchers have generated a mouse model to mimic dysmenorrhea, allowing them to investigate its causes, test potential treatments, and guide clinical care (Yang *et al.* 2015) (Fig. 1). For generating the model, non-pregnant female mice were first treated with estradiol benzoate and then injected with oxytocin to observe a writhing response as a proxy for pain response (Yang *et al.* 2015). Changes in uterine artery blood flow, uterine morphological changes, and prostaglandin changes were measured during and after this period. In this mouse model, with the injection of oxytocin, there was a decrease in uterine blood flow and an increase in oxytocin receptor, COX-2, and PGF2α. The reduced uterine blood flow caused strong and abnormal uterine contractions, and there was a strong writhing response indicative of pain (Hong *et al.* 2022). This mouse model also mimics the recurrence aspect of pain associated with primary dysmenorrhea. The model showed similar pain characteristics on the 4th and 8th days after injection, indicating pain during two successive estrus cycles in the mouse. Pain characteristics include increased writhing response, altered uterine tissue morphology, reduced uterine artery blood flow, and changes in prostaglandin levels. In addition, metabolomics analysis of the dysmenorrhea mouse model revealed significant changes in metabolites related to arachidonic acid metabolism, linoleic acid metabolism, glycerophospholipid metabolism, valine, leucine, and isoleucine biosynthesis, alpha-linolenic acid metabolism, and biosynthesis of unsaturated fatty acids (Hong *et al.* 2022). Thus, this mouse model mimics some phenotypes of the human dysmenorrhea condition and is beginning to provide insights into the mechanisms underlying dysmenorrhea and can help in identifying targets for potential new treatments (Hong *et al.* 2022).

### Limitations of mouse model of dysmenorrhea

#### Mechanisms of pain induction by oxytocin and prostaglandins

With the primary pathophysiology of human primary dysmenorrhea overwhelmingly accepted to be an overproduction of endometrial prostaglandins, a big caveat of the mouse model for dysmenorrhea is the use of excess oxytocin instead of prostaglandins to induce contractility, thus highlighting a gap in the available research models and the clinical condition of dysmenorrhea. Oxytocin-induced labor pain is partly due to ischemia and reduced blood flow to the uterine muscle (Jurna 1993). In this case, there is also stretching and distension of other pelvic organs that contribute to activation of nociceptors for pain perception. On the other hand, prostaglandins induce inflammation and make nerve endings more sensitive to pain through peripheral sensitization (Jang *et al.* 2020). Thus, the neural mechanisms of pain activation with oxytocin treatment are at least partially different from when there are high levels of systemic prostaglandins.

#### Writhing as a readout of dysmenorrhea pain

Regarding the pain types, nociceptive pain is the most common and acts as a warning signal due to the activation of nociceptors on non-neural tissue (Bonezzi *et al.* 2020). Nociceptive pain can be divided into somatic and visceral nociception. Somatic nociception originates in peripheral tissues, such as skin, muscle, and bone. Visceral nociception originates from within the abdomen or specific organs. In the mouse model for dysmenorrhea, writhing is used as an indicator for pain. While writhing is a standard preclinical endpoint, it is a non-specific behavioral indicator of visceral nociception and cannot fully capture the complex sensory and affective dimensions of dysmenorrhea experienced by patients.

#### Mechanisms of oxytocin- and prostaglandin-induced uterine contractions

A common target for both prostaglandins and oxytocin is the uterine smooth muscle, and both these modulators result in an increase in uterine contractility (Griffiths *et al.* 2006, Smith *et al.* 2007). However, the quantifiable effects of prostaglandins and oxytocin on contraction wave metrics, such as amplitude, frequency, and velocity, are not defined. Thus, these modulators may alter uterine contractility and pain differently, further highlighting the limitation of an oxytocin-induced dysmenorrhea model.

#### Differences in mouse and human uterine cycle

This mouse model mimics key clinical features of dysmenorrhea. However, mouse models cannot fully replicate the complexity of human biology with differences in terms of hormonal responses and disease progression. The uterus consistently cycles through stages, the menstrual cycle for humans and the estrous cycle for mice, and shows similar ovarian hormone fluctuations: before ovulation, estrogen levels peak and progesterone levels fall, and after ovulation, estrogen levels fall and progesterone levels rise (Hong & Choi 2018). However, the duration of each cycle is vastly different: approximately 5 days in mice and 28 days for humans. Furthermore, dysmenorrhea occurs before or during the menstrual phase of the cycle with spontaneous menstrual shedding (ACOG committee opinion no. 760: dysmenorrhea and endometriosis in the adolescent 2018) and is not part of the natural mouse estrous cycle. Since dysmenorrhea is pain associated with menstrual flow, the fact that mice (*Mus musculus*) do not naturally shed tissue is a critical biological divergence. Thus, in order to mimic dysmenorrhea, this mouse model needed to use hormone-priming followed by oxytocin treatment to generate a writhing pain response versus the natural hormone-driven recurrent menstrual period pain observed in humans.

#### Timing of onset of primary dysmenorrhea

While the onset of primary dysmenorrhea is around menarche in humans, the mouse model represents a chronic, established adult phenotype that does not coincide with the onset of estrus cycling in the mouse.

### Measuring uterine contractility in mouse and human uterus

We have limited knowledge of the dynamics of uterine contractions across the natural uterine cycle (Yang *et al.* 2015). To this effect, *in vitro* uterine contraction models for both mouse and human utilize isolated uterine strips, which are then exposed to substances such as oxytocin (Smith *et al.* 2007) and PGF2α (Griffiths *et al.* 2006) to induce contractions (Houdeau *et al.* 2003, Dodds *et al.* 2015, Dodds *et al.* 2021, Nagashima *et al.* 2023). *In vivo*, rodent uterine contractions have been quantified by inserting a balloon into the lumen to study intraluminal pressure (Robuck *et al.* 2018), attaching electrodes to the uterus to measure contractility (Talo & Kärki 1976), and using laparoscopy on anesthetized rats to record uterine contractility (Crane & Martin 1991*a*,*b*). Over the past decade, several *in vivo* and *ex vivo* spatiotemporal imaging-based methods (Zhang *et al.* 2019, Combs *et al.* 2024, Dawson *et al.* 2024) suggest that the uterine muscle is responsive to changes in the environment and contractions are dynamic throughout the rodent uterine cycle. Similarly, a non-invasive uterus imaging system has been developed using transvaginal ultrasound to visualize and quantify uterine contractility in women (Anderson *et al.* 2024). Continuing advancement of such methods will aid in a better understanding of the differences in normal cyclic contractility and how factors that cause aberrance in contractility can result in pain and dysmenorrhea.

## Current research trends in dysmenorrhea

Researchers conducted a bibliometric analysis of dysmenorrhea research. They analyzed a total of 3,407 articles and reviews published between 2000 and 2021 to identify key trends and milestones in the field. The study revealed that dysmenorrhea research has been steadily growing, indicating increasing interest and awareness in the field (Liu *et al.* 2023). Economic factors played a role in shaping research activity, with higher GDP per capita countries producing more impactful research. The analysis seems to focus primarily on English-language publications, potentially excluding valuable research published in other languages. There may also be underrepresented regions that have significant dysmenorrhea-related research but were not included due to limitations in the data sources or language bias (Liu *et al.* 2023). The study highlighted two main areas of focus: pathophysiology and public health impact. The study identified emerging research topics, such as new mechanisms like oxidative stress and the brain’s role in dysmenorrhea. The study also highlighted broader impacts of dysmenorrhea on public health, including adverse effects on quality of life and association with depression (Liu *et al.* 2023).

## Conclusions and future directions

About 45–95% of women complain of primary dysmenorrhea, but many do not report pain or pursue treatment (Iacovides *et al.* 2015). According to a study, reasons reported for not seeking treatment included assuming pain was normal and lacked treatment, being embarrassed and thinking that they can tolerate it with self-management, and having limited resources with no proper healthcare in general (Chen *et al.* 2018*b*). Among women who do seek treatment for dysmenorrhea, many end up dissatisfied with standard treatments. This could be a result of the multiple gaps that exist with dysmenorrhea.

Clinically, the study of dysmenorrhea treatments is limited by the beliefs and misconceptions of menstruation. Dysmenorrhea is underreported due to the assumption that pain is normal. Many women also refuse to seek care, believing that periods are an inappropriate subject. It often goes undiagnosed, either due to a lack of communication from the patient or due to a lack of identification from the physician (Itani et al. 2022). This lack of knowledge and awareness in the medical community ultimately plays a role in less research being conducted to treat this significant issue.

Pharmacologically, the various side effects from medications limit the availability of treatment for some. Women with stomach issues are unable to take NSAIDs, low socioeconomic status families might not be able to afford COX-2 inhibitors, and women trying to start a family cannot take COCPs or use IUDs. With only a handful of pharmacological treatments, many women have either limited options or no options for treatment at all.

From a research standpoint, current models rely on oxytocin but lack the natural prostaglandin aspect of menstruation. These non-human models also fail to include the behavioral and emotional aspects that accompany dysmenorrhea pain. More model systems resembling human biology are needed for the study of dysmenorrhea to better understand the mechanisms behind it and discover new treatments.

Despite the increased understanding of dysmenorrhea impacting the quality of life among women, there have been no recent developments in gaining a better understanding of the aberrant uterine contractions that cause dysmenorrhea. More model systems are needed for the study of dysmenorrhea and menstruation to better understand the mechanisms behind this crucial issue. Since women with dysmenorrhea have an increased sensitivity to pain stimuli, more collaboration among multidisciplinary teams of reproductive biologists, endocrinologists, geneticists, neurologists, and basic scientists is needed to help develop better treatment strategies for pain management in dysmenorrhea. There is a continued necessity for research and treatment options to alleviate the pain that women face with dysmenorrhea.

## Declaration of interest

The authors declare that there is no conflict of interest that could be perceived as prejudicing the impartiality of the work reported.

## Funding

We acknowledge support from March of Dimes Grant #5-FY20-209 and start up funds from Michigan State University.

## Author contribution statement

LK, SA, LZ, AV, and RA reviewed the literature and wrote the manuscript.

## Data availability

No new data are generated in this manuscript.
